# Evaluation of content validity and feasibility of the World Falls Guidelines’ three key questions to identify falls among older adult users of home care services in Norway

**DOI:** 10.1186/s12913-025-12606-y

**Published:** 2025-03-27

**Authors:** Rune Solli, Linda Aimée Hartford Kvæl, Nina Rydland Olsen, Therese Brovold

**Affiliations:** 1 Department of Rehabilitation Science and Health Technology, Faculty of Health Sciences, OsloMet - Oslo Metropolitan University, Pilestredet 44, Oslo, 0167 Norway; 2 Norwegian Social Research (NOVA), OsloMet– Oslo Metropolitan University, Pilestredet 44, Oslo, 0167 Norway; 3 Department of Health and Functioning, Faculty of Health and Social Sciences, Western Norway University of Applied Sciences, Inndalsveien 28, Bergen, 5063 Norway

**Keywords:** Implementation, Fall prevention, Clinical decision support, Community dwelling older people, Three key questions, Content validity, Feasibility

## Abstract

**Background:**

Falls among older adults (65 + years) is an important issue in municipal home care. Screening using the World Falls Guidelines’ three key questions (3KQ) is recommended to identify older adults at increased fall risk, but the 3KQ has not been formally tested by healthcare practitioners (HCPs) working in Norwegian municipal home care. The aim of this study was to evaluate the content validity and the feasibility of the 3KQ among HCPs in home care services.

**Methods:**

Participants were 10 multidisciplinary HCPs working in home care and in low-threshold services of Oslo, Norway. We evaluated the content validity of the 3KQ through individual think-aloud interviews. Next, feasibility was evaluated as follows: We trained HCPs in how to use the 3KQ. HCPs then screened older adults using the 3KQ during a six-week test period, and took pocket-notes of older adults’ answers. We conducted two focus groups to explore HCPs’ experiences with using the 3KQ. We analysed interview data using reflexive thematic analysis.

**Results:**

Content validity evaluation revealed that HCPs found the 3KQ easy to understand, and potentially timesaving. They experienced the tool as applicable among home care users, and it was particularly useful among new users. Still, HCPs emphasised the necessity of their training on how to best ask the questions and determine appropriate actions based on users’ responses. We identified three main themes from the feasibility evaluation: (1) Promoting awareness and action: using the 3KQ helps put falls on the agenda in municipal home care, (2) Obtaining reliable answers: integrating the 3KQ into daily practice is important, and (3) Unlocking insights: the 3KQ as a gateway to supplementary information from users. Most older adults had increased fall risk according to the 3KQ.

**Conclusions:**

The 3KQ appears feasible for Norwegian municipal home care and may be of value for HCPs who screen new users and users of low-threshold services. Integrated use of the 3KQ may enhance awareness, promote reliable answers, and provide supplementary information useful for decision-making. The study findings may benefit HCPs and managers in home care services, and other stakeholders in implementing fall prevention guidelines in primary care.

**Trial registration:**

Open Science Framework Identifier 10.17605/OSF.IO/2JFHV. Registered: 11th January 2023.

**Supplementary Information:**

The online version contains supplementary material available at 10.1186/s12913-025-12606-y.

## Background

Falls are a serious problem and increase the risk of further falls, injuries, institutionalisation, and morbidity in community-dwelling older adults 65 years or older [1]. In home care populations, the incidence of falls is between 30% [2] and 55% [3]. Previous falls, issues with gait or balance, fear of falling, higher age, and female sex increase the risk of falling [4, 5]. Globally, one in three older adults fall [6, 7] and more than 700 000 die from falls each year [1, 8]. The economic consequences of falls are considerable [9, 10], making fall prevention a critical challenge. As the world’s population continues to grow and to age while living in the community, the number of people in need of fall prevention services in municipal home care is rapidly increasing [11, 12].

Fall risk assessments and tailored fall prevention interventions can contribute to reducing falls among older adults at risk of falling [13]. Results from systematic reviews show that interventions, such as balance and strength exercise, home environment modifications and medication review, reduce the number of fallers and the number of falls [13, 14], and are cost-effective [13, 15]. Still, the implementation of summarised evidence on fall prevention into practice has been challenging [16–19]. Also, rates of falls and fall injuries have remained high the last few decades [7, 20, 21].

Older adults seldom report falls on their own [22–24] and healthcare practitioners (HCPs) do not routinely ask older adults about falls [18, 19]. Identifying individuals who would benefit from interventions begins with screening for fall risk. Current fall risk screening tools are administered as questionnaires, varying in length from one to more than twenty questions, and are often combined with a functional assessment, taking from one to more than twenty minutes to complete [25]. Examples are the Stay Independent questionnaire [26], a 12-item questionnaire including, among other risk factors, history of falls, fear of falling, perceived physical functioning, and use of fall-risk-increasing drugs, and the Three Key Questions (3KQ) [26], which are often combined with a balance assessment such as the Berg Balance Scale [27]. Lack of time is a frequently reported barrier to conducting fall prevention [28, 29], making short screening tools generally preferred for initial fall risk screening. A recent validation study suggests that shorter questionnaires, such as the 3KQ, are just as valid in predicting falls among community-dwelling older adults as more comprehensive questionnaires [30]. This aligns with the 2022 World Falls guidelines (WFG2022) recommendation that HCPs in home care services should routinely screen for fall risk among older adults using the 3KQ [16, 31]. The 3KQ questions are: 1) have you fallen in the past year?, 2) do you feel unsteady when standing or walking?, and 3) do you have worries about falling? [25]. The 3KQ originates from the STopping Elderly Accidents, Deaths, and Injuries (STEADI) initiative that provides HCPs with resources to help them integrate fall risk screening into routine practice [32, 33]. The 3KQ is a valid measure for predicting future falls [25, 34, 35], and the tool has higher sensitivity than fall risk screening tools that assess only one risk factor, e.g., focusing only on falls within the previous 12 months [25, 36, 37]. A large cohort study that included 12,346 adults age 65 or older reported fewer fall-related hospitalisations and lower healthcare expenditures after the implementation of the 3KQ and other STEADI fall risk screening and prevention strategies [38].

The 3KQ was translated into Norwegian for the Norwegian National recommendations on fall prevention [31] but content validity has not yet been evaluated in a Norwegian context. If content validity is not assessed, meaning can be lost in the translation process, which can impact validity [39]. Content validity refers to the relevance, comprehensiveness, and comprehensibility of a patient-reported outcome measure (PROM) [40], and is considered the most important measurement property of a PROM as it can affect all other measurement properties. To promote measurement properties of high methodological quality, it is also necessary to determine whether a PROM, such as the 3KQ, is acceptable to HCPs and if it is practical to implement. Acceptability (i.e., to what extent the 3KQ is judged as suitable, satisfying, or attractive to HCPs) and practicality (i.e., to what extent the 3KQ can be carried out with intended participants using existing resources and circumstances) are aspects of feasibility [41]. Feasibility is essential to evaluate before full-scale implementation [41]. Hence, the objectives of this study were to evaluate the content validity and the feasibility of the 3KQ among HCPs in Norwegian municipal home care services.

## Methods

### Design

**Fig. 1:**
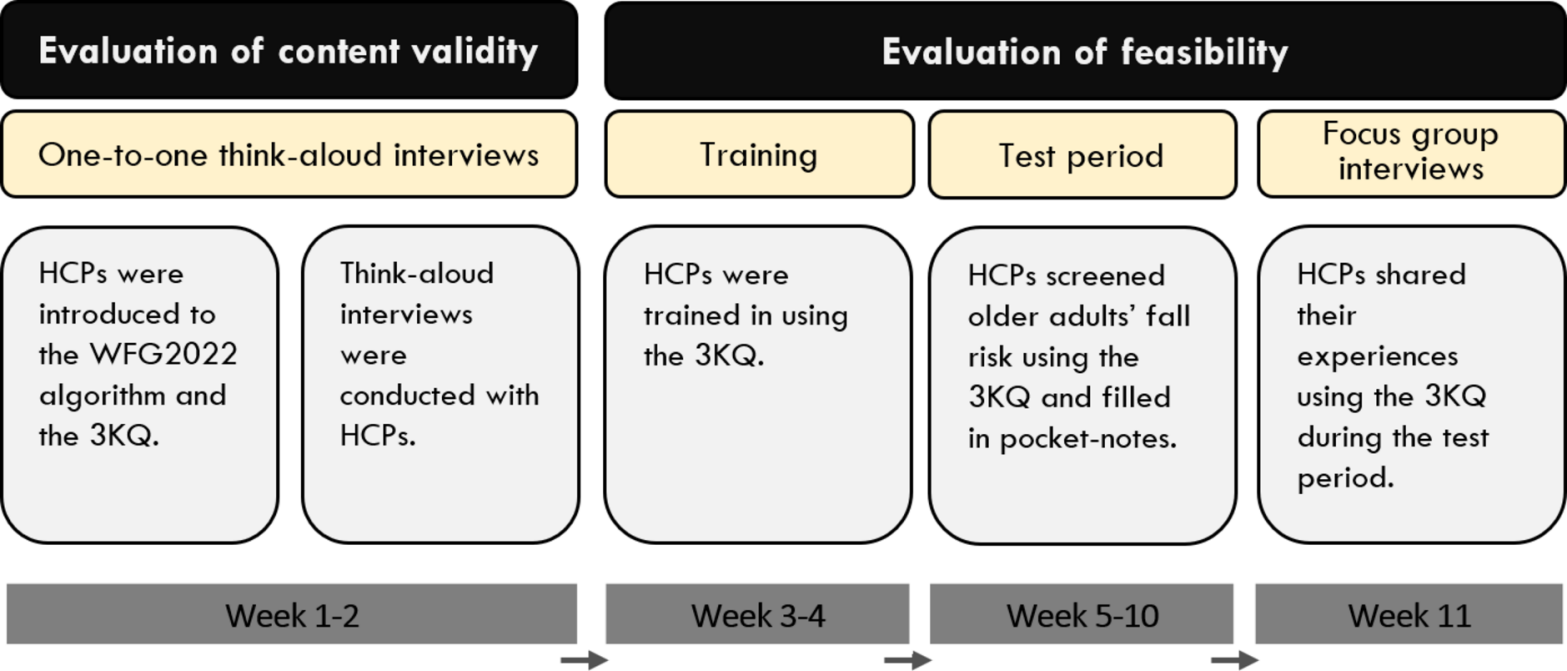
Illustration of study design. 3KQ: Three key questions; HCPs: Healthcare practitioners; WFG2022: The 2022 world falls guidelines for falls prevention and management for older adults

We evaluated the content validity and the feasibility of the 3KQ using qualitative methods. Firstly, we conducted one-to-one think-aloud interviews [42] with HCPs in home care services who had been introduced to the WFG2022 algorithm and the 3KQ [16]. Secondly, during the feasibility evaluation, we trained HCPs in how to use the 3KQ. HCPs then screened for fall risk among older adult home care recipients and noted the older adults’ answers on notes that the HCPs had with them (test period). After the test period, we conducted focus group interviews to explore HCPs’ experiences with using the 3KQ. The design of the study is illustrated in Fig. 1.

### Context

This study was conducted in the municipal home care services in one city district of Oslo, Norway. This city district consisted of three different district sections. A district section is a geographically delimited area within a city district with its own administrative unit, and is responsible for delivering home care services to its inhabitants. Home care services in this setting included home nursing, rehabilitation, and practical assistance, where multidisciplinary professionals provided services to inhabitants living in their own homes, in senior apartments, or in care homes. A care home is a facility where residents live in their own apartments and have access to round-the-clock care services, including dinner service, social activities, and exercise classes [43]. Included in home care are also low-threshold services, which are available to older adults who do not use other services, and without the need for a referral from a HCP [44]. The services are often free and may include individual follow-up, network meetings, or group therapy. The city district employs nurses, physiotherapists, occupational therapists, nursing assistants, unlicensed assistive personnel, and other professionals. The city district has experience with the implementation and systematic use of fall prevention activities, including regular home visits with comprehensive fall risk assessment and interventions. Current practice in home care does not include systematic screening for fall risk with the aim of identifying individuals who need multifactorial assessment. However, HCPs routinely visit new home care recipients to perform a multifactorial fall risk assessment following a checklist developed based on concurrent evidence [45]. Additionally, every home care recipient should receive a multifactorial fall risk assessment twice a year, or if they recently had a fall. This study is part of the research project FALLPREVENT, which focuses on implementation of evidence-based fall prevention programs in health care services in Norway [46].

### Eligibility, recruitment and participants

The head developmental nurse of the city district strategically recruited 10 HCPs who agreed to participate in the study. HCPs were eligible to participate if they had previous experience with fall prevention and were willing to participate in all parts of the study. Not all HCPs participated in all parts of the study because some were required to deliver home care services to other patients on the day of the interviews. Nine HCPs participated in the content validity evaluation and in the test period, and eight HCPs participated in the focus groups. All HCPs participated in training. The participating HCPs had background as physiotherapist (*n* = 4), nurse (*n* = 3), occupational therapist (*n* = 1), nursing assistant (*n* = 1), and unlicensed assistive personnel (*n* = 1). Six participants were women. All participants were experienced clinicians with a median 10 years of relevant work experience. The participants reported performing various fall prevention activities as part of their daily practice, including fall risk assessments and fall prevention interventions among both new and long-term users.

### Training and test period

**Fig. 2:**
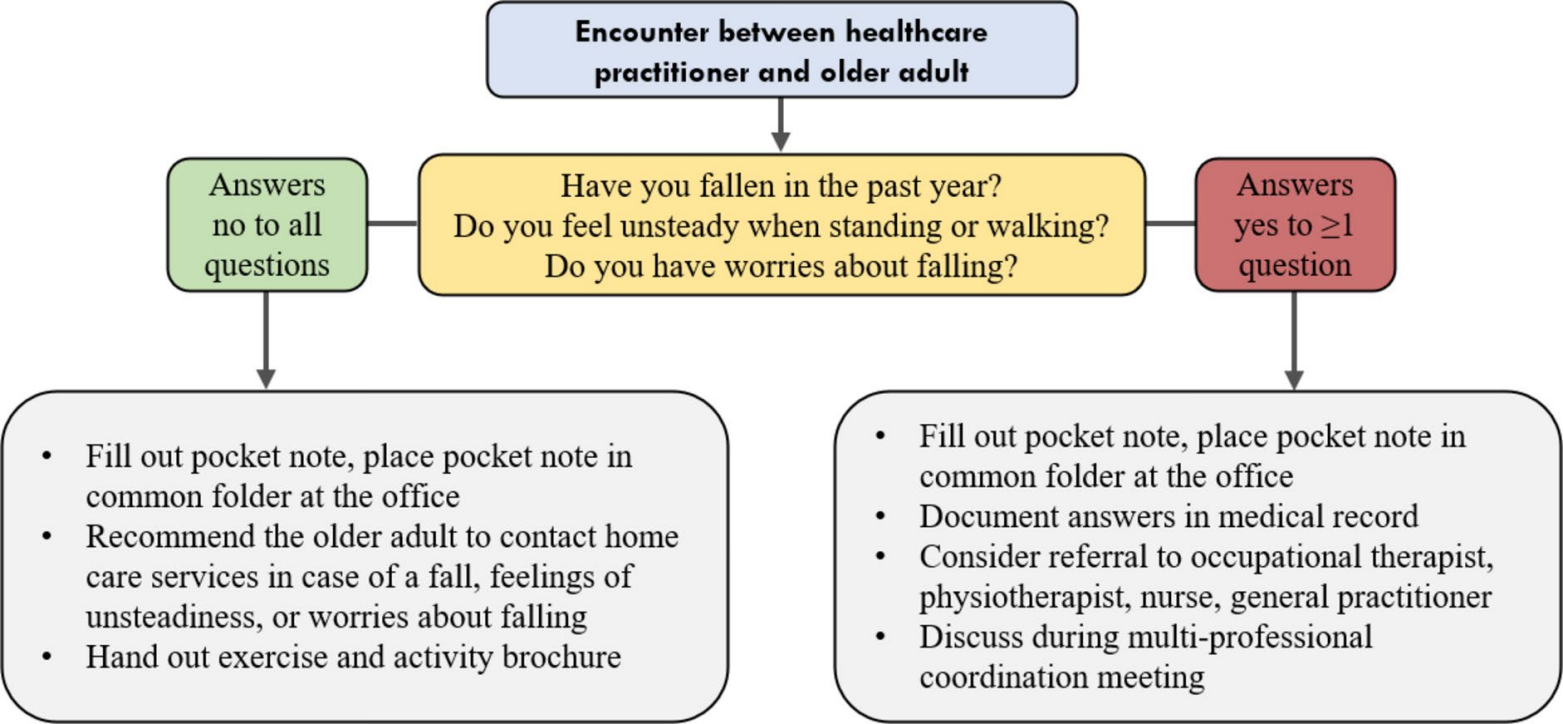
Instructions given to participating healthcare practitioners who used the 3KQ

In preparation for testing the 3KQ, participants received training by one researcher (RS). The training was held face-to-face in two groups (*n* = 2) and individually (*n* = 4). The content of the training was based on feedback received during think-aloud interviews and on discussion held in the FALLPREVENT project group. Participants were trained in how to best ask the questions to older adults and how to act on users’ responses (Fig. 2). During training HCPs were informed about the target group for the 3KQ: older adults who were 65 years or older; and who lived in their own home, in a senior apartment, or in a care home in the city district; and who received home care services or were registered users of security alarms; or were without home care services but who had a registered fall. The target group included new and long-term users, users of low-threshold services, users with cognitive impairment or dementia, and users of home nursing or of practical assistance with daily tasks.

Testing of the 3KQ took place over a six-week period from 6th March 2023 to 14th April 2023. During the test period, HCPs screened users in the target group using the 3KQ. HCPs were instructed not to inquire with the same user twice. We also instructed HCPs to screen a minimum of 15 users from each district section, and a minimum of five users of the low-threshold services. We instructed HCPs to hand out the brochure to older adults who answered ‘no’ to all 3KQ, as they were considered at low risk of falling. The brochure, designed by a physiotherapist working in the city district’s home care services, contained an overview of physical exercise and activity initiatives taking place in the city district. Users who answered yes to at least one question were considered at increased risk of future falls, and HCPs were recommended to refer these users to relevant HCPs such as a physiotherapist or an occupational therapist, and to discuss their findings during the weekly multi-professional coordination meeting. Figure 2 illustrates the 3KQ along with the instructions given to HCPs on what actions to take based on users’ responses to the questions.

### Data collection

#### Think-aloud interviews

One-to-one think-aloud interviews were held in January and February 2023. We developed a semi-structured interview guide (Additional file 1) using Consensus-based Standards for the selection of health Measurement Instruments (COSMIN)’s relevance, comprehensiveness, and comprehensibility as theoretical concepts [40]. We used the same semi-structured interview guide for all the one-to-one think-aloud interviews. During the interview, the participants were introduced to the WFG2022 algorithm and the 3KQ, which is part of the WFG2022 algorithm [16]. Subsequently, the participants were asked to think aloud, i.e., to verbalise their thinking as they read through the questions and response options of the 3KQ [42]. Then, participants were asked to discuss the following questions: (1) how do you understand the 3KQ?, (2) how do you believe the 3KQ can be used in your city district?, (3) which HCPs do you think are suitable users of the 3KQ?, and (4) who do you think are suitable target groups for the 3KQ in your city district? The one-to-one think-aloud interviews lasted from 21 to 34 min.

#### Users’ answers to the 3KQ

**Fig. 3:**
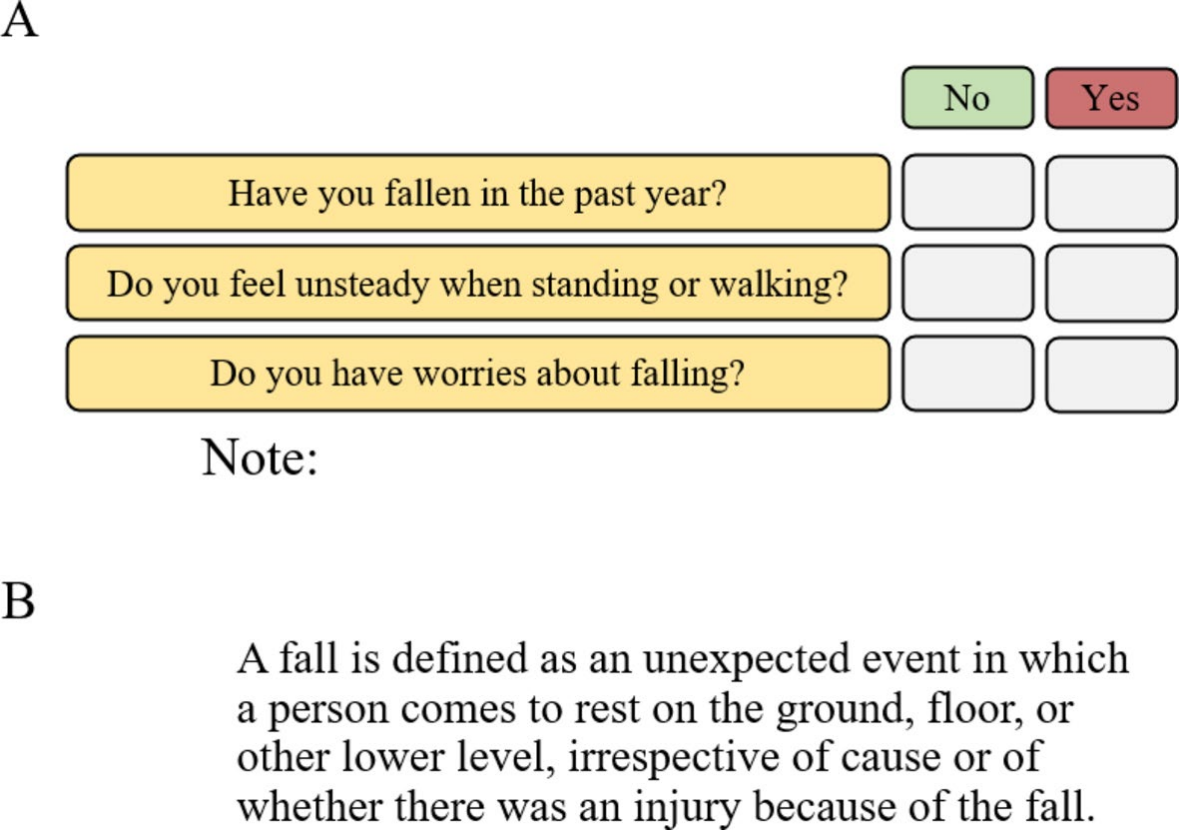
Pocket-note. **A** Frontside. **B** Backside

During the test period, HCPs recorded the users’ answers to the 3KQ on pocket-notes designed for this study (Fig. 3). The frontside included the 3KQ questions with fields for documenting responses to each question (yes/no). The backside presented the definition of a fall. One pocket-note was used for each older adult who was screened, and the pocket-notes were collected and used as data. Throughout the test period, RS kept in regular contact with participating HCPs, enquiring about their progress and whether they needed more pocket-notes or brochures.

#### Focus group interviews

Within a week after the test period, two focus group interviews were conducted with eight of the nine HCPs who participated in the test period. One focus group included two physiotherapists, one occupational therapist, and one nursing assistant, and the other included two nurses, one physiotherapist, and one unlicensed assistive personnel. We used a semi-structured interview guide (Additional file 2) to explore topics, such as experiences with using the 3KQ, and perceived usefulness, ease of use, and relevance of the tool. Both interviews were moderated by the first author (RS), with one of the co-authors being a facilitator (LAHK and TB). The two focus group interviews lasted 66 and 72 min, respectively. All interviews took place in-person in a quiet room at the home care services’ common area, and were audio recorded digitally using both a phone application [47] and a digital voice recorder.

### Data analysis

We analysed all qualitative data using reflexive thematic analysis by Braun & Clarke [48, 49]. We followed the six-step iterative method for identifying, analysing, and reporting patterns within data, known as themes [48, 49]. The themes should capture something important about the data in relation to the research questions. The following analysis process was applied to both think-aloud and focus group interview data: Analyses involved an in-depth familiarisation process with the data, including during interviewing, transcribing, and repeated reading. Two authors (RS, TB) independently coded the data by systematically working through the dataset to identify text segments that appeared relevant or meaningful. All authors participated in meetings to discuss and refine codes. Table 1 provides an example of the coding procedure. All authors cooperated on generating initial themes by compiling clusters of codes that shared a core idea relevant to the research questions. Two authors (RS, TB) then generated suggestions for main themes, which were tabulated together with the initial themes. All authors then revised the themes repeatedly. Initial themes were transferred between main themes, and each main theme was revised several times. The software HyperResearch [50] was used to aid the analysis.

**Table 1:** Example of coding procedure

Quote	Codes	Initial theme
“And if I see that someone is unsteady, I could ask ‘do you feel unsteady when standing or walking?’ And then they can get a feel for it, as opposed to just asking in conversation, because that becomes so difficult for them. It’s like ‘yes, sometimes I am, sometimes I’m not. It depends. When I’m outdoors I’m unsteady. When I’m inside, I’m not worried.” (Participant 5)	Often much more than just a “yes-” or a “no-” answer to the questions The answer to the question depends on the context	Users often provide elaborating answers

We applied different analysis approaches to the think-aloud and to focus group interviews when generating, reviewing, and refining themes. Our analysis of the think-aloud interviews was influenced by COSMIN’s aspects of content validity: relevance (considers whether the content of the 3KQ was connected to the concept being measured, i.e., fall risk), comprehensiveness (relates to if the 3KQ includes all the necessary and important aspects of the concept), and comprehensibility (pertains to how well the respondents understand the content of the 3KQ) [40]. As such, our approach was more deductive, because we used these established criteria to assess how well the data fit within this framework. However, a more inductive approach was used for the analysis of the focus groups, whereby the themes were generated without influence from a pre-existing framework. Think-aloud interviews were analysed before the feasibility evaluation, i.e., before the start of week 3. The knowledge gained from analysing the think-aloud interviews were used during the training and test period.

The authors are physiotherapists with experience from implementation research, and most had clinical experience from working with older adults and fall prevention (RS, LAHK, TB). Our preconception was that not all users in the city district received fall risk screening and interventions in line with recommendations, e.g., multifactorial fall risk assessments and interventions to high-risk participants [16]. Another preconceived notion we shared was that HCPs would find the three questions easy to understand and relevant to most user groups.

## Results

Results will be presented separately for the content validity and the feasibility evaluations, and for users’ answers during the test period.

### Content validity evaluation

Overall, the content validity evaluation revealed that HCPs found the 3KQ to be applicable to their work (relevance), covered the necessary topics for fall risk screening (comprehensiveness) and it was easy to understand (comprehensibility). HCPs believed the 3KQ was a practical tool, and potentially timesaving in municipal home care. However, Although the 3KQ may be used by several types of professionals, HCPs believed that competent use of the 3KQ requires clinical experience or training.

HCPs perceived the 3KQ as relevant to identify falls among many user groups of the municipal healthcare services. They stated that the 3KQ was likely best suited for identifying fall risk in new and cognitively intact users and users of low-threshold services. This was because new users have an unknown fall-risk and users of low-threshold services are generally well functioning and cognitively intact. HCPs perceived the 3KQ as less suited among users with cognitive impairment or a high degree of frailty. They anticipated that frail patients and cognitively impaired patients were likely to answer “yes”, and that this would jeopardise the 3KQ’s ability to discriminate fallers from non-fallers. HCPs had previous experience with asking the first question, and recognised that also the two other questions were relevant for fall risk screening and provided additional opportunity to identify users at increased risk of falling. For instance, HCPs pointed out that some users may not remember a fall within the last 12 months, however if they answer that they feel unsteady or are worried about falling, they are still considered at increased fall risk.

HCPs regarded the 3KQ as a short tool that covered the necessary topics for fall risk screening. Overall, the positive impressions stemmed from the 3KQ’s simplicity, practicality, and user-friendliness, making the tool a potential asset in their work. Furthermore, HCPs believed the 3KQ could guide resource allocation by prioritising high-risk users for comprehensive fall risk assessments, and that such assessments were unnecessary for low-risk users. However, an issue that was brought up was that HCPs and users do not always share the same notion of the term fall. For instance, in the views of some HCPs, users were reluctant to call an event ‘a fall’ if it did not involve a free fall or a hard impact. This underscores the importance of adjusting the use of the tool to the individual user. HCPs emphasised the importance of clear communication and common understanding of what a fall is between themselves and the user when addressing falls.

The participating HCPs believed that the three questions would be easy to understand for all HCPs. Still, HCPs maintained that there was a need for training in how to ask the questions and how to act on users’ responses. HCPs felt a need for understanding of how to implement the 3KQ in their practice. They indicated a need to understand not only how to pose the questions, but also how to interpret the users’ responses, and how to act on the responses from the users, enabling HCPs to take appropriate measures to prevent falls. A detailed thematic analysis of the content validity evaluation with corresponding quotes, initial themes, and main themes is available in Additional file 3.

### Feasibility evaluation

The feasibility evaluation identified three interrelated main themes: (1) Promoting awareness and action: using the 3KQ helps put falls on the agenda in municipal home care, (2) Obtaining reliable answers: integrating the 3KQ into daily practice is important, and (3) Unlocking insights: the 3KQ as a gateway to supplementary information from users. The results highlight the nuanced experiences HCPs had when testing the 3KQ in daily practice. The main themes and initial themes from the final analysis are described in Table 2.

**Table 2:** Results of the feasibility evaluation

Initial theme	Main theme
The questions put falls on the agenda	Promoting awareness and action: using the 3KQ helps put falls on the agenda in municipal home care
Refreshing with a short and intuitive tool	
The tool is better suited for certain user groups	
The questions do not always stand on their own	
The tool is used in multiple ways	Obtaining reliable answers: Integrating the 3KQ into daily practice is important
Users often provide elaborating answers	
Some users answer based on what they think will happen	
Uncertainty about how to act on the responses	
The importance of a multi-professional approach to falls	
Asking the questions help to reveal different understandings of falls among users	Unlocking insights: The 3KQ as a gateway to supplementary information from users
Comes into a position to offer the right help and information	
Users wish to maintain a façade because of stigma surrounding falls	
Can be discrepancy between healthcare practitioners’ observations and users’ answers	

#### Promoting awareness and action: using the 3KQ helps put falls on the agenda in municipal home care

The first theme captures how the HCPs valued the 3KQ as a fall risk screening tool. It also addresses how the HCPs perceived the feasibility of using the 3KQ to screen for fall risk among community-dwelling older adults using municipal home care services.

Overall, HCPs believed that the use of the 3KQ raises awareness about the importance of fall prevention. When using the 3KQ, they became more focused on assessing and preventing falls, indicating that the tool could add meaning to HCPs’ daily clinical practice through increasing their awareness of fall prevention. HCPs appreciated the 3KQ for making it easier to screen. They said that the three questions were already known to them, but that this way of structuring and asking the questions was new. HCPs were convinced that this short tool would help save time when screening for fall risk, compared to screening with the full two-page fall prevention checklist that was already in use in the city district. The 3KQ’s simplified approach provided a welcome departure from more complex protocols, enabling HCPs to navigate their tasks more efficiently without feeling overwhelmed. One HCP said:

*We already have a checklist we follow when doing these (fall risk) assessments. The 3KQ is much the same*,* only in a short version. And maybe it’s easier to ask someone about falls. It saves lots of time*,* for example on a question such as ‘are you worried about falling?’. And if you get a ‘yes’*,* you know what to elaborate on. So*,* it has been a nice addition.* (Participant 7)

During the test period, HCPs found the 3KQ to be particularly well suited for new users, users without complex challenges, and users of low-threshold services. Additionally, HCPs considered the 3KQ suited for users needing practical assistance with daily tasks and personal care, as well as those who were allocated to limited or small amounts of home care services.

*I think that these questions are well suited for* low-threshold *services*,* with those who have few (home care) services*,* or no services. And these are questions we could ask to those who are new to the services*,* to home nursing. We often bring a checklist that we use during first visits*,* and those questions could really be included in that*,* that first visit.* (Participant 5)

HCPs believed that the tool could be used to identify fall risk among new users, users without complex challenges, and users of low-threshold services. Overall, HCPs considered these groups viable target users of the 3KQ because their fall risk had not yet been assessed and the users were believed to be able to provide trustworthy answers. However, among frail users with known fall risk or dementia, the HCPs sometimes already knew the answers, or the answers were unreliable. HCPs considered the answers of some users with dementia unreliable because they forgot they had fallen and were unaware of their gait and balance issues. This required HCPs to rely on other sources of information, such as family members, caregivers, or the users’ health record, or HCPs had to make their own interpretation based on clinical expertise. This implies that the 3KQ does not provide a comprehensive fall risk assessment. Rather, HCPs considered the 3KQ practical during early identification of users in need of comprehensive fall risk assessment and potentially fall prevention interventions. One of the HCPs stated:

*And then you can move on to a comprehensive fall risk assessment and potential interventions. But to use the three questions with someone who’s been in the services over a longer period*,* I think that’s not sufficient.* (Participant 5)

HCPs appreciated the 3KQ as a time-saving initial fall risk screening tool that helped put falls on the agenda, adding value to their daily practice. HCPs also recognised its limitations with certain user groups as well as the need for additional methods to conduct a comprehensive fall risk assessment.

#### Obtaining reliable answers: integrating the 3KQ into daily practice is important

The second theme underscores the importance of competent implementation of the tool to obtain reliable answers from users. Competent implementation involves framing the conversation in a manner that promotes honest and reliable answers.

HCPs considered obtaining reliable and honest answers as a prerequisite for using the 3KQ, because knowing fall risk is the foundation for decision-making regarding fall preventive care. The answers from users can be used to decide who should be prioritised for a comprehensive fall risk assessment and fall prevention interventions. HCPs expressed various approaches to using the 3KQ. Some HCPs reported use of the questions that did not resemble integration of the tool into daily practice. During such instrumental use of the tool, the questions were asked, and the answers were interpreted without clinical judgement. In this way, screening using the 3KQ was treated as “an additional task”, and the HCPs likely did not understand or have the skills to integrate use of the tool in their practice. For instance, one HCP told the users that she needed to ask the questions as part of an ongoing project:

*I told them it’s a project. And then there were two users who wouldn’t answer because they didn’t want to participate in any project. I had to explain that it’s anonymous. ‘Can’t you just answer*,* so I can get a card?’* (Participant 4)

In contrast, a fully integrated approach entails that the HCPs applied clinical judgement and framed the questions in a manner that promoted what was perceived as more honest and reliable answers. These HCPs integrated the 3KQ with other existing tasks, and consequently, the users were not aware that the questions were part of a checklist or a project. For instance, one HCP asked the user to both stand and walk, while allowing the user time to consider if he or she felt unsteady. Another HCP chose to integrate the questions into a conversation that consisted of more than just the three questions. The following quote exemplifies this approach to the use of the 3KQ:

*When there are many questions in a big questionnaire*,* it’s more like an interview*,* and then the users pull themselves together*,* if they ‘oh*,* now*,* now I must answer correctly. Now I can’t be as weak as maybe I really am.’ But when you manage to weave the 3KQ into the conversation*,* then they forget*,* are not aware*,* so you often get more honest answers that way.* (Participant 3)

Some of the HCPs expressed uncertainty about how to act on the responses they received, which related to ambiguity in the answers from users, lack of clarity on the next steps, or uncertainty about how to interpret the information provided. Such unclarity may suggest a need for further direction or training of HCPs on how to integrate the tool into clinical practice in such a manner as to promote honest and reliable answers from users. It may also suggest a need for routines on how to act on the responses received from users.

*If you become uncertain about what to do with the answers*,* then the answers must be carried forward to others. The purpose of the questions is clear*,* even though you don’t necessarily know what to do*,* you understand that maybe something needs to be done here. And then*,* more HCPs must be included*,* or the interdisciplinary team.* (Participant 7)

According to HCPs, to obtain honest, valid, and reliable answers requires a mutual sense of trust and confidence between the HCP and the user. The HCP can promote such trust and confidence through integrated use of the questions during a conversation about falls, allowing the user sufficient time to give detailed answers, and interpreting responses using clinical judgement. The HCPs viewed this as a starting point for making clinical decisions regarding more comprehensive fall risk assessments and fall prevention interventions.

#### Unlocking insights: the 3KQ as a gateway to supplementary information from users

The third theme revealed how the 3KQ was used to obtain detailed responses beyond ‘yes’ or ‘no’ answers to the questions. Detailed responses allowed HCPs to uncover additional concerns or needs of users that they considered useful when aiming to prevent falls.

HCPs found the 3KQ practical when starting a conversation about falls, as the tool allowed users to be open about their experiences and concerns. HCPs learned about users’ perceptions about falls and falling by also asking follow-up questions based on the answers from the users. According to the HCPs, some older adults believed that falls are a natural part of ageing and cannot be prevented. HCPs also said that some users talked about not being worried about falling in their apartment, but when they were outside, they experienced worry and unsteadiness, which sometimes stopped the user from leaving their apartment. They experienced that asking the questions in an integrated manner made users more receptive to help, allowing HCPs to assist by addressing concerns about falls, and offering advice.

*When you ask them if they have fallen*,* and they say yes*,* and they confirm that they are worried*,* then it may be an opportunity to suggest interventions that may help them such as aids*,* for example. Because many users are ambivalent about the use of a walker*,* or the use of aids indoor. But if you can have a dialogue going about falls and that this (home modification or aid) may give you fewer falls*,* then maybe it’s easier to accept home modifications.* (Participant 5)

Some HCPs experienced that some users’ responses to the questions were influenced by their expectations of what the home care services would do, or by their anticipation of potential outcomes such as the installation of aids that may be visually unappealing. An example of additional insight that was unlocked by asking these questions was that some users believed that having to rely on aids was for ‘old people’, which was something the users did not want to be associated with, despite being older than 80 years. According to the HCPs, this was common, suggesting that such answers may arise from concerns regarding stigma around ageing and being dependent on aids. One of the physiotherapists suggested that, despite some users having functional limitations and needing help to perform activities of daily living, the users wanted to maintain a façade of functional independence.

*Yes*,* the second question is good*,* because I asked a lady. And then she answers (yes to feeling unsteady). And the husband says*,* ‘no you are not!’ So*,* yes*,* she was feeling unsteady. And then I find out that they don’t want any supportive devices. She was feeling unsteady*,* but this wasn’t supposed to be mentioned*,* because then*,* what is the next step? The couple expected that lots of aids should be installed in their apartment.* (Participant 4)

Even though HCPs found the 3KQ easy to understand, they believed that some users may have difficulties understanding, or may even have different understandings of a fall. This may lead to misunderstandings between users and HCPs, which HCPs considered important to avoid when seeking answers from users. All HCPs had encountered incidents they considered a fall, even though the user did not perceive them as such. Sometimes there were discrepancies between HCPs’ observations and the answers from users, making it somewhat difficult to determine what to note down as the final answer. Therefore, HCPs sometimes prioritised other sources of information than that given by the user, such as from the patient’s caregiver, the user’s journal, or from other HCPs.

Despite its short format, the 3KQ helped HCPs uncover nuanced information regarding users’ needs, differences in understanding, and concerns. This allowed HCPs to address misunderstandings and concerns and in a better way offer health care and information suited to the user’s needs.

### Results from the pocket-notes

All pocket-notes contained answers to every question. HCPs screened a total of 64 users during the test period, of which 59 answered yes to at least one question and were accordingly considered at increased risk of falls. Table 3 provides an overview of the distribution of answers to each of the questions. HCPs added comments to 20 of the 64 pocket-notes. The comments included further details on users’ answers. The comments were often related to the feeling of unsteadiness, such as “worried about falling outside, not inside”, “unsteady when outside, not when inside”, and “the user does not feel unsteady, but is very unsteady”.

**Table 3:** Answers to each of the 3KQ among users who were screened (*n* = 64)

	Yes	No
Have you fallen in the past year?	43	21
Do you feel unsteady when standing or walking?	42	22
Do you have worries about falling?	40	24
Overall, was the user considered at increased fall risk?	59	5

## Discussion

This study evaluated the content validity and the feasibility of the 3KQ among HCPs in municipal home care services of Oslo, Norway. Evaluation of content validity revealed that there was a common perception among HCPs that the 3KQ seemed relevant and comprehensible. They perceived the 3KQ to be sufficiently comprehensive for initial fall risk screening among new users, cognitively intact users, and users of low-threshold services. The feasibility evaluation revealed that HCPs experienced increased awareness of fall prevention when using the 3KQ. Integrated use of the 3KQ prompted what HCPs perceived to be honest, reliable, and detailed responses, which allowed them to gain useful supplementary information related to falls. This information was helpful for deciding how to act next, e.g., reviewing medications, making a referral, or offering assistive devices. Data from the pocket-notes revealed that 59 of 64 users had increased fall risk, and the multiple comments that were added indicated that useful additional information was gained during screening.

Results from this study indicate that HCPs experienced the 3KQ as acceptable and practical among most user groups. The short and intuitive format of the 3KQ helped them structure questions in a potentially time-saving way. This is in line with previous studies which found a complex fall prevention toolkit to be too information-packed and overwhelming [51], and that users prefer concrete instructions on fall-risk management [43]. Tiedemann et al. evaluated the feasibility of HCPs using a brief fall risk assessment tool in an Australian primary care setting [52]. Nurses, physiotherapists, and general practitioners found the brief tool acceptable and expressed that it was practical and guided the implementation of fall prevention interventions [52]. Other studies reported high satisfaction with the use of a simple checklist for fall prevention [53], and that using the 3KQ versus longer screening tools could potentially reduce the amount of time needed to screen [25]. Additionally, several projects have successfully integrated the 3KQ into routine patient care in primary care clinics [37, 38, 54]. Acknowledging these findings, it seems that HCPs generally prefer brief tools for initial fall risk screening, and they need concrete and actionable advice. Thus, while previous research has demonstrated that the 3KQ is both valid in predicting future falls [25] and implementable into routine primary care [37, 38, 54], this study builds on these findings by showing that HCPs perceive the 3KQ as an acceptable and practical fall risk screening tool. However, the 3KQ also adds additional value by raising awareness of fall prevention in home care services, and by assisting HCPs to come into a position to offer fall preventive care and information suited to the user’s needs.

Our results revealed that the 3KQ was used in multiple ways, ranging from an instrumental approach of merely asking the questions as an additional task, to a more integrated approach of integrating the questions into daily practice. These findings reveal that it is crucial to effectively communicate the questions and the reasons for asking them. In previous studies, both HCPs and older adults have expressed the importance of effective communication [51, 55–57]. As noted by Van Rhyn et al. [55], HCPs emphasised that establishing common ground was important to enhance mutual understanding and to promote adherence to fall prevention recommendations. Dickinson et al. [56] and Vincenzo & Patton [58] found that, from the perspectives of older adults, HCPs play major roles as either facilitators or barriers to older adults’ participation in fall prevention programmes. They found that how HCPs asked questions and responded to the answers and comments of older adults could facilitate or hinder older adults’ participation. As mentioned by Keuter et al. [51], older adults often prefer that their healthcare provider initiates a discussion about falls where fall prevention information is actively introduced to them, instead of relying on written information. Older adults may also need HCPs to facilitate access to fall prevention interventions, e.g., to verbally address available exercise classes, or to hand out a pamphlet outlining local fall prevention services [56]. In our study, integrated use of the 3KQ seemed to best prompt open and honest responses from users.

Integrated use can have additional advantages, beyond obtaining reliable and honest answers. As described by the HCPs, integrated use of the 3KQ allows the 3KQ to serve as a gateway to supplemental information about falls. The 3KQ helped facilitate conversation about falls and falls prevention and revealed older adults’ perceptions about falls and falling. Addressing older adults’ diverse perceptions of their fall risk and communicating that many falls are preventable may help motivate older adults to take action to prevent falls [59]. A recent meta-analysis estimated the global prevalence of concerns about falling among older adults at almost 50% [60]. According to the WFG2022, an older adult inappropriately very fearful of falling may be reluctant to increase their physical activity and follow an exercise programme [16]. Without knowledge of older adults’ beliefs or perceptions regarding these issues, HCPs cannot address them. However, our results indicate that integrated use of the 3KQ may help HCPs unlock the insight needed to offer advice and education that can be tailored to fit the perspectives, beliefs, and priorities of the older adult. Still, using the 3KQ may not necessarily answer why the patient is at increased risk for a fall. HCPs will need to assess a broader scope of clinical factors that may contribute to the older adult’s fall risk, for instance, multimorbidity, frailty, polypharmacy, or cognition [16]. Hence, initial fall risk screening should, if indicated, be accompanied by a multifactorial fall risk assessment. A systematic review reported that seven out of nine included multifactorial fall risk assessment tools adequately predict fall risk [61]. The WFG2022 highlights the necessity of a comprehensive assessment, as it allows HCPs to identify and address the various factors contributing to fall risk [16].

While this study advanced our understanding of fall risk screening using the 3KQ, there are still gaps that need to be further explored in future research. We evaluated content validity of the 3KQ tool by including the perspectives of HCPs, i.e., end users, as recommended by COSMIN [40]. However, we did not evaluate the content validity among older adults. Therefore, future research could illuminate the content validity of the 3KQ from older adults’ perspectives. Additionally, our findings suggest that HCPs find the 3KQ time-saving. However, with its high sensitivity [25, 34, 35], the 3KQ placed 59 of 64 users in this study at increased risk for falls, requiring stratification into either intermediate or high fall-risk categories and, if at high fall-risk, multifactorial assessments and interventions [16]. Thus, future studies could compare the time and resources required for multifactorial fall risk assessment using the 3KQ against other screening tools with varying levels of sensitivity. It is uncertain whether using the 3KQ for initial fall risk screening is sustainable in Norwegian municipal home care. However, Eckstrom et al. [37] successfully implemented the 3KQ in primary care, screening two-thirds of eligible patients and providing assessments and interventions to most high-risk patients. While initial enthusiasm for the 3KQ may partly explain why HCPs perceive the tool as putting falls on the agenda, participating HCPs appreciated the brevity of the 3KQ. Thus, the 3KQ’s short, user-friendly format perceived by the participating HCPs and earlier research demonstrating successful implementation [37] indicate a potential for sustainable use of the 3KQ. Nevertheless, further research is needed to determine long-term sustainability of the 3KQ as a fall risk screening tool in Norwegian municipal home care. Lastly, while HCPs were not asked to document negative screenings in the patients’ charts, it would be beneficial to document both positive and negative screenings for future reference and statistical purposes. For instance, systematically documenting both negative and positive screenings would be useful for the city district to understand how many people are screened, the proportion of older adults who screened positive or negative, and whether the proportions change according to season over the years.

### Strengths and limitations

This study has several strengths and limitations. To the authors’ knowledge, this is the first study to evaluate the content validity and the feasibility of the 3KQ among older users of municipal health care services in Norway. To ensure that HCPs’ recollection of experience with using the 3KQ was as fresh as possible, the focus group interviews were held within one week after the end of the test period. One limitation may lie in the transferability of the study results to other contexts, care levels, or users. Since testing was conducted among many with high fall risk, future testing should be conducted with a more diverse community-residing sample. However, we believe that the detailed descriptions of our methods and study context enable readers to infer contextual similarities, thereby enhancing the transferability of the study results to other yet similar contexts, recommended by Nowell et al. [62]. The recruitment of participants was based on Malterud’s information power [63], a model for ascertaining sample size that considers the study aim, sample specificity, use of theory, quality of dialogue, and analysis strategy. According to this model, the more information of relevance for the study that the participant group holds, the fewer participants are needed. If each participant can provide more valuable data, the need for a large sample size is reduced. Nine individual think-aloud interviews were held with HCPs of various backgrounds, and eight HCPs participated in focus groups. We believe these aspects as well as the use of both qualitative and quantitative data support the trustworthiness of our findings. Lastly, we believe that combining our clinical experiences with providing care to older adults, and our knowledge of the complexity of implementing new practices, was advantageous in maintaining analytic distance during data analysis [64]. Our understanding of the context allowed us to interpret the data in a nuanced way, and our understanding of the complexity of implementing new practices has made us open to findings that may contradict our own beliefs or previous experiences.

## Conclusions

The 3KQ that is recommended by the World Falls Guidelines appears acceptable and practical for HCPs working in Norwegian municipal home care services. HCPs found the 3KQ relevant, sufficiently comprehensive, and comprehensible for initial fall risk screening of new and cognitively intact users. HCPs were most familiar with the question “have you fallen in the past year?” Still, HCPs perceived that questions two and three added value by being able to identify risk of falling among users with no history of falls. Older adults’ responses may be influenced by their expectations of what the home care services may do, such as installing visually unappealing aids. However, integrated use of the 3KQ seemed to facilitate open, reliable, and detailed answers from users. This helped HCPs gain insight into the perspectives, beliefs, and priorities of older adults, all important topics to address when co-designing a fall prevention intervention together with the older adult.

Finally, these results indicate that the 3KQ can serve as an acceptable and practical part of the comprehensive WFG2022 falls assessment and management algorithm, potentially benefitting HCPs who screen for fall risk. The 3KQ may also be used to facilitate open dialogue about falls and falls prevention with the older adult. Hopefully the benefits extend to users of home care services, who may be offered appropriate fall prevention services according to their fall risk.

## Supplementary Information

Additional file 1. Think-aloud interview guide.

Additional file 2. Focus group interview guide.

Additional file 3. Content validity evaluation results.

## Data Availability

The datasets generated and analysed during the current study are not publicly available to protect the confidentiality of participants, but are available from the corresponding author on reasonable request.

## Abbreviations

3KQ: Three Key Questions
CDS: Clinical Decision Support
STEADI: STopping Elderly Accidents, Deaths, and Injuries
TSD: Services for Sensitive Data
WFG2022: The 2022 world guidelines for falls prevention and management for older adults: a global initiative
