# Supplementary material for: Evaluation of content validity and feasibility of the World Falls Guidelines’ three key questions to identify falls among older adult users of home care services in Norway
Source: BMC Health Serv Res. 2025 Mar 27;25:444. doi: 10.1186/s12913-025-12606-y (PMC11948927; doi:10.1186/s12913-025-12606-y)
Supplement: Supplementary file 1 — Additional file 1. Think-aloud interview guide. [file 12913_2025_12606_MOESM1_ESM.docx]

**Additional file 1. Think-aloud interview guide**

Initially, the moderator will inform the interviewee what will happen. The interviewee has received information about the project and has signed informed consent. Prior to the interview we will discuss privacy with the interviewee and inform the interviewee that they must avoid mentioning individuals by their name and characteristics that can be tied to individuals.

**Part 1. Think-aloud**

1.1 Information about and presentation of the world falls guidelines 2022 and the 3KQ tool used to identify older adults with increased fall risk

RS: “The first part of the interview is dedicated to think-aloud about the 3KQ tool, created to identify older adults with increased fall risk. Later, I want to hear your thoughts about the tool, and please think aloud and verbalise your thoughts. Please mention what part of the tool you are talking about when you comment, so that I understand what part you are talking about. For example, you can mention which question or which box you are talking about. First I want to present background information on the tool. In September of 2022, the World guidelines for falls prevention were published. The guidelines highlight the fact that the world’s population is aging, and that falls and fall injuries are increasingly common. Therefore, fall prevention is a crucial challenge. Healthcare practitioners are recommended to routinely ask about falls during their encounters with older adults. All older adults who encounter healthcare practitioners, no matter the reason for the encounter, should be asked a minimum of once each year if they have 1) fallen during the past year, 2) feel unsteady when standing or walking, and 3) worry about falling. In the guidelines there is a figure showing the recommendations step by step. I will now start the audio recording and show you the figure.”

RS places a paper copy of the WFG2022 algorithm on the table in front of the interviewee and gives the interviewee time to look at the figure.

RS: “You are not required to read all of it. This figure is from the new guidelines, and I am showing this to you for some background information on where the tool we will test comes from. The figure is meant to give a step-by-step description of the fall prevention process with identification of who is at low, moderate, and high fall risk, assessments of the individual’s risk factors for falling, individually tailored interventions to prevent falls, and follow-up of the older adult. The tool that is the focus of this interview has been created from the first part of this figure.”

RS points to the first part of the WFG2022 algorithm where the case-finding part of the algorithm is located, and places a copy of the 3KQ on the table in front of the interviewee. The interviewee is given time to look at the 3KQ.

RS: “This is what the tool looks like. When there is an encounter between a healthcare practitioner and an older adult, the healthcare practitioner will ask: Have you fallen in the past year? Do you feel unsteady when standing or walking? And – Do you have worries about falling? If the older adults answers no to all questions, it is recommended to provide education regarding fall prevention, physical activity, and exercise. You can also hand out a brochure with written information. If the older adult answers yes to at least one question, a referral for further fall risk assessment is to be made.

**Guiding questions**

- General questions
  - How do you understand the tool as a whole?
  - Is the content of this tool entirely new to you, or do you already have questions that you use to identify older adults with increased fall risk?
  - Do you regularly ask about falls in your city district?
  - How do you understand the question?
  - Will the question be understandable to older adults?
  - How difficult do you think it will be to get to know the tool – that is to say, to remember everything by heart and understand the use of the tool?
  - Does it make sense to provide verbal or written information if the older adult answers no to all questions?
  - Does it make sense to make a referral for a fall risk assessment if the older adult answers yes to at least one question?
  - Do you see some challenges with using the tool in the home care services?
- The first question
  - How do you understand the word fall?
- Second question
  - How do you understand the word unsteady?
- Third question
  - How do you understand the word worry?

**Part 2: Use of the tool**

RS: “Now I want to hear your thoughts on how this tool can be used in the city district?”

- What can be done to have this tool become used routinely in the city district?
- How often should the tool be used in the same person/user?
- I what situations should the tool be used?
- In what situations should the tool not be used?
- Is there a need for training in use of the tool – if so, what kind of training?
- Do you need to bring the figure along during your workday, or will you be able to remember it by heart?
- What do you think about having a small note that you can use to write down the answers?

**Part 3: Who can use the tool?**

RS: “Now I want to hear your opinion on who can use this tool to identify older adults with increased fall risk.”

- Which professions can use this tool?
- Are there some professions or persons this tool is better suited for than others?
- Are there some professions or persons who cannot use this tool?

**Part 4: Who can be a suitable target group?**

RS: “Now I want to hear your opinion on who can be a suitable target group for the questions in the tool here in the city district. This will help us plan the target group during the test period.”

- Who should be asked these questions in this city district?
- For instance, should those with a known fall risk be asked the questions, or only those with an unknown fall risk?
- What about users with nursing care versus users with practical assistance?
- What about users of the safety alarm versus users who do not have the safety alarm?
- What role does age play regarding what users the questions are suitable for?
- What role does level of functioning play regarding what users the questions are suitable for?
- What role does disease play regarding what users the questions are suitable for?
- What about living arrangement?
