# Supplementary material for: Evaluation of content validity and feasibility of the World Falls Guidelines’ three key questions to identify falls among older adult users of home care services in Norway
Source: BMC Health Serv Res. 2025 Mar 27;25:444. doi: 10.1186/s12913-025-12606-y (PMC11948927; doi:10.1186/s12913-025-12606-y)
Supplement: Supplementary file 2 — Additional file 2. Focus group interview guide. [file 12913_2025_12606_MOESM2_ESM.docx]

**Additional file 2. Focus group interview guide**

Moderator:

Welcome. How nice that you have the opportunity to participate, we appreciate it. You signed informed consent during the first interview, so now we won’t collect a new consent form. Participation is voluntary, and you can withdraw at any time without consequences. We want to conduct an audio recording of the interview so we can write down what was said afterwards. We will use quotes, and you may be able to recognize them, but we will make sure that it will be completely anonymised.

A focus group interview is a conversation between you where you have the opportunity to share experiences and discuss among yourselves. Here, no answers or opinions are correct or wrong. In this focus group interview we want to listen to your experiences with use of the tool (the three questions). The purpose of the tool is to identify older adults with increased fall risk. We welcome you to share examples, but we ask you not to share sensitive information. We have set aside 75 minutes, and then we’ll see how much time we need. If this still sounds alright to you, we’ll start the audio recording.

(The moderator turns the audio recording on on both units).

**Preliminary questions: Getting started**

1. Now we have started. First, will you please start by presenting yourselves with your first name, professional background, and what role you play in fall prevention?
2. Now you have participated in a project where you have tested these questions. How did you experience that?

**Part 1: About experiences with use of the tool**

1. How did you experience using the three questions?
   1. Please give examples of situations where you used the tool.
   2. Do these questions make sense for you to ask?
2. Can you talk about situations where you did not use the tool?
3. Can you talk about how you experienced the content of the training prior to the start of the test period?
4. Based on your experiences, who do you think can use the tool or ask these questions? Please explain why.

**Part 2: About perceived usefulness**

1. Can you talk about how useful the tool is in your setting / part of home care services?
   1. What worked well with regard to using the tool?
   2. What didn’t work well with regard to using the tool?
2. How did the users experience having these questions asked?

**Part 3: About perceived ease of use**

1. How easy or how complex do you it is to use the tool?
2. Can you talk a little bit about how you initiated the conversation about falls? Provide examples.
3. Can you talk a little bit about how the users experienced answering these three questions?
   1. If you experienced a user who didn’t understand, what did you do?
4. What do you think is the next step after you have asked the three questions?

**Part 4: About the relevance of the tool**

1. What are your thoughts about how relevant the tool is in your part of home care services?
2. What is needed for you to continue using the tool and asking the questions to the users?

**Wrapping up**

Now we are at the final part of the interview and we want to give you the opportunity to add thoughts or to provide other feedback you think may be relevant.
