# Supplementary material for: Evaluation of content validity and feasibility of the World Falls Guidelines’ three key questions to identify falls among older adult users of home care services in Norway
Source: BMC Health Serv Res. 2025 Mar 27;25:444. doi: 10.1186/s12913-025-12606-y (PMC11948927; doi:10.1186/s12913-025-12606-y)
Supplement: Supplementary file 3 — Additional file 3. Content validity evaluation results. [file 12913_2025_12606_MOESM3_ESM.docx]

**Additional file 3. Content validity evaluation results**

| **Quotes** | **Code clusters** | **Initial themes** | **Main themes** |
| --- | --- | --- | --- |
| “And then we’re back to, can everyone ask these questions? Yes, everyone can. But can you ask them to everyone? That’s not certain. And then it’s not certain that everyone can ask the questions, because it depends on who you ask them to.” (Participant 6)  “And maybe people who have, have worked for a long time can ask the questions. One could look a bit at work experience. Perhaps you don’t assign this to the new untrained worker who is starting.” (Participant 2) | Can be used by many professions, but requires clinical experience | Appropriate fall risk screening tool for HCPs in home care services | **A relevant tool for a range of HCPs, suitable to many user groups** |
| “Because we do have a lot of people in the healthcare system who, yes, know Norwegian well, and some who don’t know Norwegian that well. But these questions, they are very easy to understand for all healthcare practitioners.” (Participant 1) | The questions are appropriate even for HCPs who do not have Norwegian as first language |  |  |
| “Yes, the questions are understandable for those who don’t have a diagnosis related to cognitive impairment or dementia.” (Participant 10)  “I think this is a tool we should use, maybe especially with those who are entirely new to the care services, to get going, to screen for fall risk.” (Participant 6)  “My opinion is that one should focus on those who are entirely new to the services and who we don’t know that well.” (Participant 1)  “And then there’s the open health and wellness program in the city district that typically see users who don’t already have home care services. The open health and wellness program that can focus even more on that screening for fall risk.” (Participant 4) | The 3KQ is most suited for new users, users of the open health and wellness program, and cognitively intact users | Suitability of diverse groups of users in home care services |  |
| “You can’t ask a person with advanced dementia about this because you won’t get a clear answer. And people who have, yes who, who perhaps have early cognitive impairment. So you won’t get proper answers from everyone.” (Participant 6)  “One can ask the questions, but I can’t always trust the answers. So, the target group is, in a way, everyone, but, I’d say that those with significant cognitive impairment would, in a way, you can’t always trust their answers.” (Participant 4) | Challenging to apply to users with cognitive impairment |  |  |
|  |  |  |  |
| “The questions cover quite well, I think. Everyone proceeds to a fall risk assessment anyway, if there is a ‘yes’ to one of the questions.” (Participant 10) | The questions cover what they are supposed to cover | Three questions that cover important topics for identifying falls | **The 3KQ seems sufficiently comprehensive for initial fall risk screening** |
| “I think it’s very easy to use the tool. It’s very clear what we healthcare practitioners are supposed to do. And we, everyone who works in home care, have been through something similar, so it will be very easy to accept that tool and to implement and use it with patients.” (Participant 10)  “I think all can use the 3KQ tool. The questions are so straightforward, it’s just yes no, also professionals and non-professionals. And you don’t do a big assessment, you ask, ask three questions, with a yes or no answer. Everyone can do that.” (Participant 1)  “So, this specific tool contains three short, easy questions, that are very easy to understand. And I think that then we should absolutely utilise it.” (Participant 1) | Three short feasible questions | Implementing the tool in a hectic clinical workday seems feasible |  |
| “I think that that prioritising fall prevention activities in those with the highest risk is very wise. Because we don’t have the capacity to perform a comprehensive fall risk assessment for each individual older adult. That one can, to those who aren’t supposed to get a comprehensive fall risk assessment, can give supervision and advice.” (Participant 5)  “And if we have some entirely new users who answer no to everything, yes, well, then it’s okay. Then we don’t need to conduct a comprehensive fall risk assessment and spend unnecessary time on it. Because we have limited resources.” (Participant 1) | It is unnecessary to perform a comprehensive fall risk assessment in all users |  |  |
|  |  |  |  |
| “These are three relatively simple questions to remember.” (Participant 8)  “Yes the older adult will understand the second question, when our users say they have to lean on furniture, walls, that they have to lean on a walker or other aids, then they’re unsteady.” (Participant 10)  “The first two questions are very, very easy to understand. Are you worried? I would’ve said ‘are you worried or afraid of falling?’ Maybe, to simplify a bit, also because we meet quite a few with a minority language, so ‘worried’ can be a bit more difficult than ‘afraid’.” (Participant 9) | Simple and straightforward questions | Three easy questions to identify falls | **The 3KQ questions are clear, but use of the tool requires training of HCPs and individual adaptation to users** |
| “I think that there’s always a need for training in what to do when out with the users. Eh, maybe also something how to, how to ask the questions, and when. Not that you just throw it out there. Because that’s what some people need. That is, communication, simply put. I’ve been out on assessments where healthcare practitioners don’t tell the user why they ask what they ask, they just ask. Eh, that, that yes, it’s more that, you must explain a bit also, ‘the reason that I ask about this is because …’, give some background, that ‘I ask this question because.’” (Participant 6) | To understand how to use the questions, healthcare practitioners need training | Promoting shared comprehension through training of HCPs |  |
| “… I think that what one needs training in is, and to agree on, is how to proceed if it’s ‘no’. And the same with, what do you do if it’s a ‘yes’, and how do you proceed. And not necessarily, it won’t be training in the tool, but how do you document and ensure follow-up, with a ‘yes’. That’s what you need training in.” (Participant 9) | There is a need for a routine on what to do with the answers that might be nuanced |  |  |
| “Yes, I know that the meaning of a fall is involuntarily down on the floor. So, as a healthcare practitioner, I think that as long as you’re on the floor because you couldn’t avoid it, then it’s a fall. I think the users, at least some of them, think more like, yes, they physically fall, but like ‘bam, hard impact’, that is, that they actually hit the ground. So, there’s a bit of a difference in definitions.” (Participant 2) | Older adults can have a different understanding of “fall” | Perceptions of “fall” vary among older adults and understanding these nuances improves clinical encounters |  |
| “It’s important that the healthcare practitioners who are with the users the most ask these questions.” (Participant 6)  “At the same time, it could be a bit problematic if you don’t know the user. And the task of screening for falls lands on me that one day, and I haven’t been with the user for four months, kind of. It’s better that there’s one who knows the user” (Participant 4) | Advantageous to know the user for a good dialogue |  |  |

3KQ: Three key questions; HCP: Healthcare practitioner
